# The Effect of Mechanical Tongue Cleaning on Oral Malodor and Tongue Coating

**DOI:** 10.3390/ijerph19010108

**Published:** 2021-12-23

**Authors:** Ha-Na Choi, Young-Sik Cho, Jung-Wan Koo

**Affiliations:** 1 Department of Dental Hygiene, Jeonju Kijeon College, Jeonju 54989, Korea; callichn@naver.com; 2 Department of Dental Hygiene, Namseoul University, Cheonan 31020, Korea; cyoungs2101@gmail.com; 3 Department of Occupational and Environmental Medicine, Seoul St. Mary’s Hospital, College of Medicine, The Catholic University of Korea, Seoul 06591, Korea

**Keywords:** mechanical tongue cleaning, oral malodor, toothbrush, tongue coating, tongue scraper

## Abstract

Background: Mechanical tongue cleaning is an important oral hygiene procedure; it is known that a significant cause of volatile sulfur compounds (VSCs), a major component of bad breath, is due to the bacteria coating the tongue. This study was conducted to identify the effect of mechanical tongue cleaning on reducing bad breath and tongue coating. Methods: Various mechanical tongue-cleaning methods were studied, including removing tongue coating using a toothbrush, removing tongue coating using a tongue scraper, and removing tongue coating using a toothbrush and a tongue scraper together. The results were as follows. Results: First, the organic bad breath measurement value after cleaning the tongue significantly decreased in the group using only the toothbrush, the group using only the tongue scraper, and the group using both the toothbrush and the tongue scraper. However, there was no difference between the groups. Second, after cleaning the tongue, the measured values of the tongue coating in the values of WTCI (Winkel’s tongue coating index) and Qray view were significantly reduced in all three groups, and there was no difference between the groups. Third, the gas measurement value in the oral cavity using a machine significantly decreased only the H_2_S value of the group using the tongue scraper immediately after the mechanical tongue cleaning. Conclusions: From these results, it can be confirmed that mechanical tongue cleaning is effective at reducing bad breath and tongue coating. However, in this study, there was no difference in the reduction effect according to the tools (groups) used for mechanical tongue cleaning. It can therefore be seen that wiping accurately from the rear of the tongue to the front is more effective at reducing bad breath and tongue coating.

## 1. Introduction

Bad breath refers to breath odors originating in the mouth that others find unpleasant. Since this can play an important role in social communication, managing bad breath is important from both a social and a health perspective. In developed countries, 8–50% of people continue to experience bad breath [1] and suffer from bad breath [2]. In 1972, McNamara identified that the cause of bad breath was bacteria in the mouth that made the ‘volatile odoriferous solecule’ [3], public interest in bad breath began to increase, and bad breath clinics and commercial products began to appear. According to data from the National Oral Health Survey in Korea, the use of dental floss among oral hygiene products related to bad breath increased from 7.10% in 2003 to 14.65% in 2010, and the use of mouth rinse increased from 6.48% in 2003 to 21.64% in 2010 [4,5].

Bad breath caused by intra-oral causes, that can only be effectively reduced by oral hygiene management, account for about 85 to 90% of cases. Most instances of bad breath are caused by oral tissue diseases and pathological conditions due to tongue fur, periodontal disease, and dry mouth [6,7].

The occurrence of bad breath is closely related to the proteolytic anaerobic bacteria present in the oral cavity. Anaerobic bacteria act on amino acids (methionine, cysteine, cystine) containing sulfur in the oral detachment epithelial cells, leukocytes, saliva, and food to produce volatile sulfur compounds (VSC) hydrogen sulfide (H_2_S), methyl mercaptan (CH_3_SH), and dimethyl mercaptan ((CH_3_)_2_S). In a study by Hinodes, the main components of bad breath were reported as volatile sulfur compounds (VSCs) including hydrogen sulfide (H_2_S), methyl mercaptan (CH_3_SH), and dimethyl sulfide ((CH_3_)_2_S) [8]. Tangerman and Winkel have provided evidence that CH_3_SH and H_2_S are the main causes of bad breath [9].

Anaerobic bacteria present in the periodontal pocket and on the dorsum of the tongue can decompose sulfur-containing amino acids to form volatile sulfur compounds (VSCs) [3,10]. In healthy people, food and bacteria accumulated in the dorsum of the tongue are reported to be the most important causes of bad breath [11,12]. It has also been reported that tongue fur can be the cause if there is a difference in VSC levels, even though there are no differences in clinical conditions such as oral hygiene, missing teeth, and caries [13].

Methods of preventing or treating bad breath include self-treatment, including tooth brushing, tongue cleaning (remove tongue coating), and mouth rinsing, or expert treatment such as scaling, periodontal treatment, preservation treatment, and prosthesis treatment. Oral malodor caused by oral factors can be effectively reduced by dental hygiene intervention. Mechanical tongue cleaning has become important as an oral hygiene procedure, as VSC components have been identified as the main cause of bad breath [14]. The results of a study on clinical trials on oral odor treatment in periodontal disease patients reported that non-surgical periodontal treatment (scaling, tooth polishing), oral health education (tooth brushing, interdental management), and tongue cleaning were the primary approaches to reducing oral odor [15]. In a study on the effect of reducing bad breath through tongue cleaning, tongue coating was found to be the main cause of bad breath; therefore, it is necessary to manage tongue coating [12]. According to a systematic review on effective tongue coating management methods, tongue scrapers are more effective at reducing bad breath than toothbrushes [16].

Through previous studies, has been found that tongue coating acts as the main cause of bad breath. Current forms of tongue cleaning reduce bad breath, but existing studies have not conducted a quantitative investigation of tongue coating. The measurement method of inspection has mainly been used as the method for measuring the amount of tongue coating; the limitation of this is that measurement results may vary depending on the inspector. Therefore, it is necessary to quantitatively measure tongue coating, an important factor that causes bad breath. QLF (quantified light-induced fluorescence)-D is a device that detects red fluorescence from a metabolite called porphyrin, secreted from bacteria present in the oral cavity. A digital camera with a built-in special light source and filter can be used to continuously photograph general white light source images and QLF fluorescence images. Red fluorescence can check images in areas with bacterial membranes, and tongue coating can be quantitatively measured by automatically analyzing only the areas where bacterial membranes are deposited [17]. This study aims to confirm the effect of mechanical tongue cleaning on the reduction of tongue coating and bad breath, through the quantification of tongue coating using QLF-D. Using a toothbrush and tongue scraper, which are commonly used to remove tongue coating to remove bad breath, we intend to find an efficient bad breath management method by observing the effects of reducing bad breath and tongue coating.

## 2. Materials and Methods

### 2.1. The Subject of the Study

This study was conducted on 56 people who agreed to participate in the bad breath management program, conducted by the Department of Dental Hygiene at N University in Cheonan, from 1 May to 3 August 2012. The sample size was calculated using the measurement value of halitosis as the primary evaluation variable. The significance level was 5%, and the statistical power was 80%. Based on the reference results, the expected difference between the evaluation variables was calculated as 40%. As a result, the sample size was 45. Considering that the dropout rate was 20%, we assigned 18 people to each group.

The selection criteria were a result of 2 or more on the Organic Test, and 4 or more on the tongue coating test (WTCI); those who did not receive any periodontal treatment during the 6 months prior to the study were selected. As a result of the oral examination, at least one area with a periodontal cyst depth of 4 mm or more, severe tooth decay (C3 or more), general diseases related to bad breath (condensation, chronic rhinitis, asthma, lung disease, bronchial diseases, gastrointestinal diseases, kidney disease, oral dryness, etc.) and pregnant women, were excluded from this study. Of the 56 subjects, 37 were male and 19 were female, and the average age was 23.9 ± 5.4 years (male: 24.5 ± 6.3, female: 22.7 ± 3.0).

This study was conducted after receiving approval (MC12EASI0022) from the ‘Institutional Bioethics Review Board of Catholic University’.

### 2.2. Methods

#### 2.2.1. Study Design

This study was a randomized controlled trial. A different mechanical tongue-cleaning method was performed for each group. The experimental group assignment was independently performed by the Data Coordinator. The experimental group assignment was carried out once the preparations for intervention were completed, after obtaining consent to participate in the study from the subjects. The examiner did not know the tongue-cleaning method in each group. Before and immediately after tongue cleaning, the halitosis value and tongue coating value for each group were measured.

#### 2.2.2. Experimental Treatment: Mechanical Tongue Cleaning

The experimental treatments in this study were classified into a group (*n* = 20) using a toothbrush, a group (*n* = 18) using a tongue scraper, and a group (*n* = 18) using a toothbrush and a tongue scraper together to remove tongue coating. The groups either used a toothbrush and tongue scraper a total of 10 times from the rear to the front of the tongue, or a toothbrush and tongue scraper 5 times each. After wiping the tongue, all the subjects were instructed to rinse the mouth lightly with water. In this study, a toothbrush (#412, Dental Mania, Seoul, Korea) was used as a tool for tongue cleaning, and cleaning products (Placon Tongue Cleaner, Oksan Preden, Daegu, Korea) were used as a tongue scraper.

#### 2.2.3. Measurement Tool

In this study, the oral examination, microbial examination, tongue coating examination, and bad breath examination were performed by a trained examiner.

- Oral examination:

For the oral examination, a dental caries, periodontal condition and oral biofilm examination were performed. Teeth were examined for decay (DT), missing teeth (MT), fillings (FT)—decayed, missing or filled teeth (DMFT)—and bleeding on probing (BOP). The periodontal screening and recording system (PSR) was used to establish the participants’ periodontal status. In the oral biofilm examination, the O’Leary plaque index was measured.

- Tongue coating score:

For the tongue coating score, a value was obtained by scoring the tongue coating using WTCI (Winkel’s tongue coating index), QLF (quantified light-induced fluorescence)-D, and Qray view. WTCI is a method of visually examining the amount of tongue coating by an examiner. The subject’s tongue is extended, and the back of the tongue is divided into three parts at the front, and three parts at the rear. The six parts are scored from 0 to 2 points (0 = no TC, 1 = thin TC, 2 = thick TC). QLF-D uses a digital camera with a built-in special light source and filter to continuously photograph general white light source images and QLF fluorescence images, automatically analyze the tongue coating deposition area with software, and display the amount of tongue coating as a percentage (%). Qray view is used for tests such as oral biofilm, tooth decay, and tongue coating using hand-type Q-ray generators and goggles with built-in filters. After checking the red fluorescence in the area with the biofilm using a Q-ray viewer, the back of the tongue is divided into six equal parts and scored in the same way as the WTCI measurement method. The range of measured values is the same as in WTCI.

- Microbiological test:

For the microbial test, the BANA–Zyme test was performed. When bad breath is caused by periodontal disease, *Treponema denticola* and *Porphyronas gingivalis* are microorganisms that exert an important influence on bad breath. This is a method for culturing and testing bacteria (*P. gingivalis*, *T. denticola*, *T. forcemia*) that are known to cause bad breath, and are related to periodontal disease in tongue coating collected from the back of the tongue.

- Oral malodor test:

For the bad breath test, organoleptic test and intra-oral gas measurement using a machine were performed. In-oral gas measurements using machines were performed using Oral Chroma (CHM-1, Abilt, Osaka, Japan), BB Checker (mBA-21, Plustech, Daejeon, Korea), and Attain (mBA-400, Plustech, Daejeon, Korea).

1. The organoleptic test is a sensory test that is scored based on the examiner’s perception of bad breath. One trained examiner examined all the subjects. Before conducting the study, two inspectors conducted the organoleptic test on 20 people to evaluate the degree of agreement between investigators and within investigators, and each showed a 95% agreement rate. The organoleptic test was performed by Rosenberg’s method [18]. The subjects were seated upright on the dental chair, kept their lips closed for 3 min, and then counted from 1 to 11, at which point the examiner evaluated the smell at a distance of 10 cm from the subject’s mouth. The evaluation scores were classified into: 0, no odor; 1, suspected odor; 2, weak but certain odor; 3, medium odor; 4, strong odor; and 5, severe odor. Measurements were taken at intervals of 10 min or more for each measurement in consideration of the inspector’s adaptability to smell.
2. Intra-oral gas measurements using machines were carried out according to the manufacturer’s manuals using Oral Chroma (CHM-1, Abilit, Osaka, Japan), BB Checker (mBA-21, TAIYO instrument Inc., Japan), and Attain (mBA-400, TAIYO instrument Inc., Japan). Oral Chroma (CHM-1, Abilit, Osaka, Japan) is a form of equipment that separates and measures the relative amounts of hydrogen sulfide (H_2_S), methyl mercaptan (CH_3_SH), and dimethyl sulfide ((CH_3_)_2_S), which are volatile sulfur compounds (VSC) known to cause bad breath. The BB Checker (mBA-21, TAIYO instrument Inc., Japan) is a device that measures the subject’s intra-oral gas, exhaled gas, and odor. Attain (mBA-400, TAIYO instrument Inc., Japan) is an in-oral ammonia gas meter that amplifies and observes the ammonia produced by bacteria, by placing elements into the subject’s oral cavity to predict the amount of bacteria.

The organoleptic, tongue coating, and intra-oral gas tests were performed before and immediately after intervention. The subjects were instructed not to use cosmetics or perfumes on the morning of the day of measurement, and not to consume any food or perform oral hygiene for 2 h before the measurements. In addition, they were instructed not to clean the tongue for a week before the measurements.

### 2.3. Data Analysis Method

The data were analyzed using SPSS 18.0 (PASW Statistics). The Wilk–Shapiro test was performed for normal distribution by group and, as a result, the rest of the variables except for the Qray view and BB values did not follow the normal distribution. Therefore, to compare oral health statuses, a cross tab analysis was performed with the Kruskal–Wallist test, the organic measurement values and tongue-coating measurements (WTCI, QLF-D), and the intra-oral gas measurements (H_2_S, CH_3_SH, (CH_3_)_2_S, Attain) between each group, before and immediately after intervention. In addition, a Wilcoxon-signed rank test was performed to obtain the mean differences between the organic breath measurements, tongue-coating measurements (WTCI, QLF-D), and intra-oral gas measurements (H_2_S, CH_3_SH, (CH_3_)_2_S, Attain), before and after intervention. The Qray view and BB values were used to perform a paired-t test to obtain the mean difference before and after intervention, and a one-way ANOVA was performed to compare the mean between the groups.

## 3. Results

### 3.1. Oral Health Status before the Intervention

As a result of checking the oral health status of the subjects before the intervention, there were no significant differences between the groups on the DMFT, periodontal status, oral hygiene, and BANA tests, as shown in Table 1 and Table 2.

### 3.2. Organoleptic Test Score before and after Intervention

The organoleptic test scores showed statistically significant differences in the toothbrush group (*p* = 0.001), the tongue scraper group (*p* = 0.000), and the toothbrush and tongue scraper group (*p* = 0.001), before and after intervention.

There was no difference in the organoleptic test score by group after intervention (*p* = 0.635) (Table 3).

### 3.3. Tongue Coating Score before and after Intervention

#### 3.3.1. Tongue Coating Score (WTCI)

WTCI showed statistically significant differences before and after intervention in the toothbrush group (*p* = 0.000), the tongue scraper group (*p* = 0.000), and the toothbrush and tongue scraper group (*p* = 0.000). There was no difference in WTCI by group after intervention (*p* = 0.445) (Table 4).

#### 3.3.2. Tongue Coating Score (QLF-D)

The QLF-D values showed no difference in scores before and after intervention in the toothbrush group (*p* = 0.083) or the toothbrush and tongue scraper group (*p* = 0.328). The tongue scraper group showed statistically significant differences in scores before and after intervention (*p* = 0.018). There was no statistically significant difference in QLF-D values for each group after intervention (*p* = 0.551) (Table 5).

#### 3.3.3. Tongue Coating Score (Qray View)

The Qray view values showed statistically significant differences before and after intervention in the toothbrush group (*p* = 0.000), the tongue scraper group (*p* = 0.001), and the toothbrush and tongue scraper group (*p* = 0.000). There was no difference in Qray view values for each group after intervention (*p* = 0.805) (Table 6).

### 3.4. Measurement of Gas in the Oral Cavity before and after Intervention

#### 3.4.1. H_2_S

There was no difference in H_2_S values before and after intervention in the toothbrush group (*p* = 0.050) or the toothbrush and tongue scraper group (*p* = 0.798), although there was a statistically significant difference in the tongue scraper group (*p* = 0.005). After intervention, the H_2_S values did not differ for each experimental group (*p* = 0.377) (Table 7).

#### 3.4.2. CH_3_SH

There was no statistically significant difference in CH_3_SH values before and after intervention in the toothbrush group (*p* = 0.056), the tongue scraper group (*p* = 0.110), or the toothbrush and tongue scraper group (*p* = 0.799). There were no statistically significant differences in CH_3_SH values for each group after intervention (*p* = 0.322) (Table 8).

#### 3.4.3. (CH_3_)_2_S

There was no statistically significant difference in (CH_3_)_2_S values before and after intervention in the toothbrush group (*p* = 0.074), the tongue scraper group (*p* = 0.167), or the toothbrush and tongue scraper group (*p* = 0.586). There were no statistically significant differences in (CH_3_)_2_S values for each group after intervention (*p* = 0201) (Table 9).

#### 3.4.4. BB Value

The BB value differed in the toothbrush group before and after intervention (*p* = 0.048). There was no difference before and after intervention in the tongue scraper group (*p* = 0.801) or the toothbrush and tongue scraper group (*p* = 0.611). There were also no statistically significant differences in BB values for each group after intervention (*p* = 0.912) (Table 10).

#### 3.4.5. Attain

There was no difference in Attain values before and after intervention in the toothbrush group (*p* = 0.669), the tongue scraper group (*p* = 0.259), or the toothbrush and tongue scraper group (*p* = 0.677). There were also no statistically significant differences in the Attain values for each group after intervention (*p* = 0.642) (Table 11).

## 4. Discussion

Among the causes of bad breath in the oral cavity, tongue coating is known to be the most important [19]. Tongue coating is characterized by a white appearance of the tongue due to residue, white blood cells, microorganisms (fungus and bacteria), and food residue eliminated between the papillae filiforms [20]. The protein hydrolysis of these microorganisms in the oral cavity contributes to the production of volatile sulfur compounds that release odors in the mouth [21]. Mechanical tongue cleaning as an oral hygiene procedure is important, because it is known that the significant cause of volatile sulfur compounds (VSCs), a major component of bad breath, is the bacteria in tongue coating [14]. Improving oral hygiene, including tongue cleaning and wiping the back of the tongue using a toothbrush or tongue scraper to remove food residue, cells, and bacteria present between the tongue papilla, can reduce oral bacteria [22,23].

This study was conducted in order to determine the reduction effect of bad breath and tongue coating through mechanical tongue cleaning, and to find out the difference in the reduction effect according to the mechanical tongue-cleaning method.

As a result, the measurement of the organoleptic test scores after tongue cleaning was significantly reduced in both the group using only a toothbrush, the group using a tongue scraper only, and the group using a toothbrush and a tongue scraper together. This was consistent with the results of previous studies that have suggested that tongue cleansing is effective at reducing bad breath [12]. However, there was no difference in bad breath reduction between the groups. Seemann’s study reported that using a tongue cleaner, a combination of a toothbrush and a tongue scraper, is more effective in reducing bad breath than using regular toothbrushes, or a tongue scraper [24]. A study by Pedrazzis reported that a tongue coating remover is more effective at reducing bad breath than a toothbrush [25]. According to Seemann’s study, tongue cleaners are more effective than toothbrushes because toothbrushes are slightly smaller than tongue cleaners and tongue scrapers [24], and the toothbrush is simply a preprocessing process, that loosens the residue deep in the tongue. Given the results of these studies, it was expected that there would be a difference in the effect of reducing bad breath according to the tongue-cleaning method, but there was no difference in this study. Although the tool used for tongue cleaning was different from that used in previous studies, the tongue cleaning was performed by the same person in this study, and the tongue cleaning was repeated from the rear to the front of the tongue, the cleaning method in previous studies. Based on the results of tongue cleaning using the same product for each group, and the reduced bad breath after intervention, it would be difficult to regard the tools or methods used as essentially different. Previous studies on tongue cleaning have reported that tongue scrapers are more effective at reducing bad breath than toothbrushes, and that patients prefer tongue scrapers [25]. However, we explained the limited effectiveness of the tongue scraper by reporting that, although the tongue scraper is more effective at reducing VSC, there is less effective difference than the toothbrush; the duration of the effect is also short, and incorrect use causes injury [26]. Therefore, from the results of this study alone, regardless of the differences between the mechanical tongue-cleaning method, it can be seen that accurate tongue cleaning from the rear to the front of the tongue is effective at reducing organic bad breath.

Among the measurements of tongue coating, the values of WTCI and Qray view decreased significantly in all three groups after tongue cleaning, and there was no difference for each group. The tongue-coating measurement is also considered to be effective in reducing tongue cleaning, regardless of the difference between the three tools when used properly and accurately.

The measured value of gas in the oral cavity using a machine significantly decreased only the H_2_S value of the group using the tongue scraper immediately after tongue cleaning. A study by Outhouse reported that tongue scrapers are more effective at reducing volatile sulfur compounds (VSCs) than toothbrushes [26]. H_2_S is a major component of volatile sulfur compounds, and this study also showed its significant reduction when using tongue scrapers.

This study confirms the effect of reducing bad breath and tongue coating of mechanical tongue cleaning. Organic bad breath decreased in all three groups through mechanical tongue cleaning, and the values of WTCI and Qray view decreased. As for the intra-oral gas measurement, H_2_S was significantly reduced in the group using the tongue scraper.

The organoleptic test of bad breath is scored based on the examiner’s perception of bad breath. In reality, it is difficult to accurately measure odors with machines, so organoleptic tests are known as the “Gold Standard” of bad breath tests [21]. From the results of this study, it can be confirmed that mechanical tongue cleaning is effective at reducing bad breath and tongue coating. However, in this study, there was no difference in the reduction effect according to the tools (groups) used for mechanical tongue cleaning, so it can be seen that wiping accurately from the rear of the tongue to the front is more effective at reducing bad breath and tongue coating. Since bad breath is a painless symptom, its severity is likely to be underestimated and, compared with other oral diseases, the frequency of visiting hospitals for treatment is low. However, in a modern society that values individual image, it is becoming increasingly important to manage bad breath, which can negatively affect interpersonal relationships. It can be seen that removing tongue coating in an accurate and correct way is a more effective way to manage bad breath than simply using a toothbrush or tongue scraper.

The limitation of this study is that the examination for systemic disease in the screening process of the study subjects was conducted only by questioning the subjects. The failure to select patients through more accurate and objective results may have affected the research results.

Most of the studies on the effect of reducing bad breath reported the effect of toothpaste, mouth rinse, and gum to reduce bad breath [27,28,29,30]. Compared with the fact that tongue coating is known to be an important cause of bad breath, randomized clinical trials comparing the reduction of bad breath by mechanical tongue cleaning are rarer than might be expected. In this respect, this study can be seen as meaningful in that it features a randomized clinical trial that confirms the effect of reducing bad breath and tongue coating according to three mechanical tongue-cleaning methods. However, research is needed to confirm not only the reduction effect immediately after intervention, but also longer-term effects, and research should be conducted through a more accurate screening process for systemic diseases that may affect bad breath in the future.

## 5. Conclusions

This study was conducted in order to assess the effect of reducing bad breath and tongue coating through mechanical tongue cleaning, and to assess the difference in the reduction effect according to mechanical tongue-cleaning methods. Among the mechanical tongue-cleaning methods were: removing tongue coating using a toothbrush; removing tongue coating using a tongue scraper; and removing tongue coating using a toothbrush and a tongue scraper together. The results were as follows.

First, the organic bad breath measurement value after cleaning the tongue mechanically significantly decreased in the group using only the toothbrush, the group using only the tongue scraper, and the group using both the toothbrush and the tongue scraper. However, there was no difference between the groups.

Second, after cleaning the tongue mechanically, the measured values of tongue coating using the values of WTCI (Winkel’s tongue coating index) and Qray view were significantly reduced in all three groups, and there was no difference between the groups.

Third, the gas measurement value in the oral cavity using a machine significantly decreased only the H_2_S value of the group using the tongue scraper immediately after the mechanical tongue cleaning.

From these results, it can be confirmed that mechanical tongue cleaning is effective at reducing bad breath and tongue coating. However, in this study, there was no difference in the reduction according to the tools used for mechanical tongue cleaning, so it can be seen that wiping accurately from the rear of the tongue to the front is more effective at reducing bad breath and tongue coating than the tool itself. Therefore, bad breath can be reduced simply by removing the tongue coating that causes bad breath, using a toothbrush or a tongue scraper. It can be seen that removing tongue coating in an accurate and correct way is an important way to manage bad breath.

## Figures and Tables

**Table 1:** Oral health status of the subjects (DMFT, BOP, PCR).

.		Toothbrush (*n* = 20)			Tongue Scraper (*n* = 18)			Toothbrush and Tongue Scraper (*n* = 18)			*p*-Value ^†^
		M (SD) *	Med **	Range	M (SD) *	Med **	Range	M (SD) *	Med **	Range	
DMFT	DT	1.45 (2.48)	0.00	0–10	0.94 (1.21)	0.50	0–4	3.22 (4.53)	1.00	0–17	0.180
	MT	0.15 (0.49)	0.00	0–2	0.33 (1.03)	0.00	0–4	0.28 (0.58)	0.00	0–2	0.581
	FT	4.00 (2.64)	5.00	0–8	4.39 (4.60)	4.00	0–14	3.56 (3.67)	2.50	0–11	0.784
	DMFT	5.45 (3.91)	5.50	0–15	5.67 (4.92)	4.00	0–14	7.06 (4.72)	7.00	0–18	0.549
Periodontal status	BOP	1.45 (2.59)	0.00	0–9	1.06 (1.63)	0.00	0–6	3.56 (5.19)	1.00	0–17	0.397
Oral hygiene	PCR	49.64 (23.51)	53.15	10–91	47.37 (23.02)	47.40	13–85	56.82 (29.65)	69.40	11–90	0.476

* Values are reported as the mean (standard deviation); ** values are reported as the median; ^†^ *p*-value are determined by Kruskal–Wallis test.

**Table 2:** Oral health status of the subjects (PSR, BANA) *n* (%).

		Toothbrush (*n* = 20)	Tongue Scraper (*n* = 18)	Toothbrush and Tongue Scraper (*n* = 16)	Δ	*p*-Value *
PSR	0	11 (19.6)	5 (8.9)	8 (14.3)	5.587	0.232
	1	6 (10.7)	4 (7.1)	4 (7.1)		
	2	3 (5.4)	9 (16.1)	6 (10.7)		
	total	20 (35.7)	18 (32.1)	18 (32.1)		
BANA	0	10 (17.9)	8 (14.3)	12 (21.4)	1.946	0.378
	1	10 (17.9)	10 (17.9)	6 (10.7)		
	total	20 (35.7)	18 (32.1)	18 (32.1)		

* *p*-values are determined by chi-square test.

**Table 3:** Organoleptic test score.

	Intervention	Baseline			Immediately after Cleaning			Δ	*p*-Value ^†^
		M (SD) *	Med **	Range	M (SD) *	Med **	Range		
Organoleptic test	Toothbrush	2.85 (0.75)	3.00	2–4	1.95 (0.95)	2.00	0–4	0.90 (0.64)	0.000
	Tongue scraper	2.94 (0.87)	3.00	2–5	1.94 (1.11)	2.00	0–5	1.00 (0.59)	0.000
	Toothbrush ^††^ Tongue scraper	2.89 (0.76)	3.00	2–4	2.17 (0.92)	2.00	1–4	0.72 (0.58)	0.001
	Total	2.89 (0.78)	3.00	2–5	2.02 (0.98)	2.00	0–5	0.88 (0.61)	0.000
	*p*-value ^††^	0.974			0.635				

* Values are reported as the mean (standard deviation); ** values are reported as the median; ^††^ *p*-value are determined by Wilcoxon signed rank test; ^†^ *p*-values are determined by Kruskal–Wallis test.

**Table 4:** Tongue coating score (WTCI).

	Intervention	Baseline			Immediately after Cleaning			Δ	*p*-Value ^†^
		M (SD) *	Med **	Range	M (SD) *	Med **	Range		
WTCI ^∥^	Toothbrush	6.15 (2.35)	6.00	4–12	4.25 (2.27)	3.00	2–11	1.90 (1.12)	0.000
	Tongue scraper	6.61 (2.50)	6.50	4–12	4.72 (2.02)	4.00	3–9	1.89 (1.37)	0.000
	Toothbrush and Tongue scraper	7.33 (2.33)	7.00	4–12	4.72 (2.68)	3.50	1–11	2.61 (1.29)	0.000
	Total	6.68 (2.40)	7.00	4–12	4.55 (2.30)	3.00	1–11	2.13 (1.28)	0.000
	*p*-value ^††^	0.272			0.445				

^∥^ WTCI: Winkel’s tongue coating index. * Values are reported as the mean (standard deviation). ** Values are reported as the median. ^††^ *p*-value are determined by Wilcoxon signed rank test. ^†^ *p*-value are determined by Kruskal–Wallis test.

**Table 5:** Tongue coating score (QLF-D).

	Intervention	Baseline			Immediately after Cleaning			Δ	*p*-Value ^†^
		M (SD) *	Med **	Range	M (SD) *	Med **	Range		
QLF-D ^∥^	Toothbrush	20.65 (28.18)	7.00	0–86	17.10 (26.86)	3.50	0–85	3.55 (8.64)	0.083
	Tongue scraper	24.50 (28.87)	13.50	0–84	17.56 (24.59)	1.50	0–70	6.94 (12.99)	0.018
	Toothbrush and Tongue scraper	21.56 (22.40)	14.00	0–78	20.76 (24.37)	12.00	0–73	−0.19 (16.93)	0.328
	Total	22.20 (26.40)	12.00	0–86	18.38 (24.95)	5.00	0–85	3.57 (13.04)	0.004
	*p*-value ^††^	0.760			0.551				

^∥^ QLF-D: Quantified light-induced fluorescence-D; * values are reported as the mean (standard deviation); ** values are reported as the median; ^††^ *p*-values are determined by Wilcoxon signed rank test; ^†^ *p*-values are determined by Kruskal–Wallis test.

**Table 6:** Tongue coating score (Qray view).

	Intervention	Baseline			Immediately after Cleaning			Δ	*p*-Value ^†^
		M (SD) *	Med **	Range	M (SD) *	Med **	Range		
Qray View	Toothbrush	7.50 (2.52)	8.00	4–12	5.50 (2.54)	5.00	2–11	2.00 (1.30)	0.000
	Tongue scraper	6.83 (2.15)	7.00	4–11	5.22 (1.90)	5.00	3–9	1.61 (1.69)	0.001
	Toothbrush and Tongue scraper	8.06 (2.01)	8.00	5–12	5.72 (2.32)	5.00	3–11	2.33 (1.78)	0.000
	Total	7.61 (2.19)	8.00	4–12	5.61 (2.26)	5.00	2–11	1.98 (1.59)	0.000
	*p*-value ^††^	0.273			0.805				

* Values are reported as the mean (standard deviation); ** values are reported as the median; ^††^ *p*-values are determined by paired *t*-test; ^†^ *p*-values are determined by one-way ANOVA.

**Table 7:** Portable sulfide monitor score (H_2_S).

	Intervention	Baseline			Immediately after Cleaning			Δ	*p*-Value ^†^
		M (SD) *	Med **	Range	M (SD) *	Med **	Range		
H_2_S ^∥^	Toothbrush	59.90 (90.37)	14.00	0–318	34.75 (66.43)	0.00	0–274	25.15 (54.97)	0.050
	Tongue scraper	91.44 (105.16)	50.50	0–315	25.94 (63.86)	0.00	0–260	65.50 (92.18)	0.005
	Toothbrush and Tongue scraper	64.83 (50.92)	68.50	0–167	58.83 (83.12)	0.00	0–287	6.00 (75.21)	0.798
	Total	71.62 (85.05)	44.00	0–318	39.66 (71.49)	0.00	0–287	31.96 (77.61)	0.003
	*p*-value ^††^	0.378			0.377				

^∥^ H_2_S: Hydrogen Sulfide; * values are reported as the mean (standard deviation); ** values are reported as the median; ^††^ *p*-values are determined by Wilcoxon signed rank test; ^†^ *p*-values are determined by Kruskal–Wallis test.

**Table 8:** Portable sulfide monitor score (CH_3_SH).

	Intervention	Baseline			Immediately after Cleaning			Δ	*p*-Value ^†^
		M (SD) *	Med **	Range	M (SD) *	Med **	Range		
CH_3_SH ^∥^	Toothbrush	7.85 (13.28)	2.50	0–51	3.35 (8.63)	0.00	0–33	4.50 (10.59)	0.056
	Tongue scraper	11.67 (15.51)	1.50	0–45	6.83 (22.18)	0.00	0–94	4.83 (22.94)	0.110
	Toothbrush and Tongue scraper	7.78 (11.14)	1.50	0–37	8.83 (14.59)	0.00	0–49	−1.06 (17.75)	0.799
	Total	9.05 (13.30)	2.50	0–51	6.23 (15.78)	0.00	0–94	2.82 (17.49)	0.084
	*p*-value ^††^	0.900			0.322				

^∥^ CH_3_SH: methyl mercaptan; * values are reported as the mean (standard deviation); ** values are reported as the median; ^††^ *p*-values are determined by Wilcoxon signed rank test; ^†^ *p*-values are determined by Kruskal–Wallis test.

**Table 9:** Portable sulfide monitor score ((CH_3_)_2_S).

	Intervention	Baseline			Immediately after Cleaning			Δ	*p*-Value ^†^
		M (SD) *	Med **	Range	M (SD) *	Med **	Range		
(CH_3_)_2_S ^∥^	Toothbrush	15.25 (22.82)	10.00	0–105	8.20 (12.62)	1.00	0–49	7.05 (16.70)	0.074
	Tongue Scraper	16.61 (27.81)	7.50	0–117	6.17 (9.88)	1.00	0–29	10.44 (28.43)	0.167
	Toothbrush and Tongue Scraper	18.39 (19.37)	15.00	0–84	17.94 (25.21)	10.00	0–81	0.44 (32.16)	0.586
	Total	16.70 (23.16)	12.00	0–117	10.68 (17.55)	2.00	0–81	6.02 (26.13)	0.032
	*p*-value ^††^	0.434			0.201				

^∥^ (CH_3_)_2_S: dimethyl sulfide; * values are reported as the mean (standard deviation); ** values are reported as the median; ^††^ *p*-values are determined by Wilcoxon signed rank test; ^†^ *p*-values are determined by Kruskal–Wallis test.

**Table 10:** Portable sulfide monitor score (BB value).

	Intervention	Baseline			Immediately after Cleaning			Δ	*p*-Value ^†^
		M (SD) *	Med **	Range	M (SD) *	Med **	Range		
BB value	Toothbrush	67.40 (19.11)	69.00	21–95	60.65 (19.11)	62.50	30–96	6.75 (14.26)	0.048
	Tongue Scraper	59.11 (11.36)	58.50	33–76	58.39 (13.55)	60.00	29–81	0.72 (11.95)	0.801
	Toothbrush and ^†^^†^ Tongue scraper	58.83 (20.92)	55.50	24–101	60.67 (21.82)	65.00	25–93	−1.83 (15.02)	0.611
	Total	61.98 (18.37)	61.50	21–101	59.93 (18.20)	61.50	25–96	2.05 (14.06)	0.279
	*p*-value ^††^	0.262			0.912				

* Values are reported as the mean (standard deviation); ** values are reported as the median; ^††^ *p*-values are determined by paired *t*-test; ^†^ *p*-values are determined by one-way ANOVA.

**Table 11:** Portable sulfide monitor score (Attain).

	Intervention	Baseline			Immediately after Cleaning			Δ	*p*-Value ^†^
		M (SD) *	Med **	Range	M (SD) *	Med **	Range		
Attain	Toothbrush	24.60 (18.14)	20.00	5–88	26.65 (22.12)	20.00	7–90	−2.05 (15.67)	0.669
	Tongue scraper	21.39 (33.73)	13.50	0–150	24.00 (31.31)	20.00	0–130	−2.61 (10.25)	0.259
	Toothbrush and ^†^^†^ Tongue scraper	27.94 (25.12)	20.00	5–90	25.83 (23.31)	20.00	5–107	2.11 (13.54)	0.677
	Total	24.64 (25.83)	20.00	0–150	25.54 (25.32)	20.00	0–130	−0.89 (13.36)	0.831
	*p*-value ^††^	0.208			0.642				

* Values are reported as the mean (standard deviation); ** values are reported as the median; ^††^ *p*-values are determined by Wilcoxon signed rank test; ^†^ *p*-values are determined by Kruskal–Wallis test.
